# Dr. Chapman's Elements of Therapeutics and Materia Medica

**Published:** 1823-06-01

**Authors:** 


*Elements of Therapeutics and Materia Medica. To which are prefixed two Discourses on the History and Improvement of the Materia Medica, originally delivered as Introductory Lectures.* By N. Chapman, M. D. Professor of the Institutes and Practice of Physic, and Clinical Lecturer in the University of Pennsylvania: President of the Academy of Medicine of Philadelphia. &c. Two volumes, 8vo. second editition. Philadelphia, 1821.

The Practice of Medicine may be said to consist of two separate trains of investigation and study—*first*, to ascertain the nature, seats, and causes of diseases, including anatomy, physiology, and pathology—*secondly*, to discover and apply the means of obviating or curing diseases when they occur, including chemistry, materia medica, and clinical experiments and observations. Although it might appear, at first sight, that in the latter class of studies (being merely the observation of facts and occurrences) there could be little cause for discrepancies of opinion, yet, with the exception of chemistry, we have in the said class, a most ample field for theory, hypothesis, contradictory doctrines, and clashing facts. The modus operandi of medicines, for instance, and the share they take in the removal of diseases, have afforded bones of contention innumerable, in the medical world, down to the present moment. It is now, indeed, pretty generally acknowledged that we can rarely, if ever, predict the operation of a medicine, from an examination of its sensible qualities—many being very powerful in their effects on the human frame which are apparently inert or insipid—while others, of very striking sensible qualities, may be taken in large doses, without any adequate consequences. But while the operation of medicinal substances is thus reduced to a mere matter of observation upon trial, their *modus agendi* presents a very wide field for speculation—a field in which the author of the work before us has ranged very freely, and not always idly or uselessly. Our American brethren pride themselves, in some measure, on their emancipation not only from the political but the medical dogmas of their European progenitors; and in such a science as ours, it is not difficult to suppose that such liberty sometimes becomes a little licentious. We are not disposed, however, to find fault with our transatlantic brethren on this account, as we are no advocates for tame submission to the yoke of any other authority than truth and experience.

Professor Chapman is a man who evidently thinks for himself, and who is capable of thinking well, on many occasions. We do not consider him entirely devoid of prejudices, nor unapt to form what we might term *ardent* opinions occasionally—a leaning to which we think we can perceive in most of the American authors.*

Having been called unexpectedly, in the year 1813, to the chair of materia medica in the University of Pennsylvania, he had but little time to prepare for his lectures, of which he only delivered three courses before he was translated to another chair. His former pupils pressed him to print his lectures, and the work before us is the result of this request. The first edition appeared in 1817, and the present has undergone a careful revision, and we may suppose considerable improvements. We shall now endeavour to give some account of the work to our cisatlantic brethren.

The first *discourse* is on the *history* of the materia medica, which we pass over as a matter of course; and the second also, with the exception of the passage already quoted in a note.

The first *section* of Dr. Chapman's work is on the *modus operandi* of medicines, and here we have "the vigorous sallies of speculative genius," but whether truth is revealed in "a burst of light of celestial brightness," we cold-blooded critics of the old world must not be in too great a hurry to decide. A warm controversy is carried on beyond the Atlantic, between the solidists and the humoralists, our author being at the head of the former party.

It is conceded on all sides that the operation of medicines does not depend on the common laws of matter, but on a

---

* We shall give an extract from Dr. Chapman's second discourse, in the general bearing of which we coincide with our author; but the language will convey some idea of what we meant by *ardent opinions*.

"Experience, by which I understand that species of knowledge which is acquired by frequent investigation of the same object, has been much insisted upon, as the only guide to successful practice in medicine. Were this well founded, it would only be necessary diligently to attend the receptacles of the sick, and any well-trained nurse might be brought to excel the most enlightened and regularly educated physician. Let this principle be once admitted, and our practice becomes a blind routine, without reason or reflection—and the profession, instead of being studied as heretofore, as a science, will be considered merely as a mechanical art, and exercised only as a low and vulgar trade. Can we consent to this degradation? As well might we compare the mere flutterings of the meanest and the most grovelling bird, with the bold and well sustained flight of JOVE's own imperial eagle, *as those slow processes of a vulgar intellect by which facts are collected or observed,* with the vigorous sallies of speculative genius, which seize truth, as it were, by intuition, and reveal it in a burst of light of celestial brightness." 54.

principle incident to vitality. "Medicamenta non agunt in cadaver," is a scholastic maxim. But what is vitality? Our author, we think, wisely abstains from entering deeply into this subject. Of the various doctrines respecting the vital principle, only one appears to him to be at all well founded—and this it is which presumes that every animated body, animal or vegetable, is endowed with *a primordial principle of life*, resident in the ova of animals, and the seed of plants, constituting the power by which the various organs are moulded, developed, and perfected, and by which the animal economy is afterwards defended against the action of mechanical and chemical laws. In this we perfectly agree with our author;—and in the greater part of the following positions we are also inclined to coincide.

"*Located*, perhaps, in the highest degree, among the digestive and assimilative organs, it enables them to change, or destroy the qualities of the substances exposed to their operation, without sustaining in return the slightest injury or change. It would hence really appear, that instead of matter, whether aliment, drink, or medicine, acting on the living system, as is commonly imagined, it is, on the contrary, the living system which operates on these matters. But, such is the case only, when the vital energies are in a vigorous and healthy condition. Different, indeed, is the result, where, from debility or other imperfection, the vital organs are rendered unfit to act upon substances, or of resisting the action of substances on the system. Whatever is taken into the stomach under such circumstances, preserves its properties unaltered, or undergoes the same sort of alteration, which it would do out of the body, or beyond the sphere of the vital powers. Each article in this state obeys the order of its affinities, and the changes which occur are purely chemical. Common matter now acting by its own laws, the system, being thus languid and decayed, sinks under an attack it cannot repel, and the processes of fermentation and putrefaction ensue, which, if not timely arrested, become the precursors, as well as the causes of death and destruction." 60.

We can hardly accord with our author in all parts of the above quotation. Suppose the material introduced into a healthy stomach be a glass of brandy. We feel first a glow of heat in the stomach, and next a mental excitement or exhilaration of spirits. Does our author suppose that no "*change*" is produced in the nervous or vascular systems of the stomach and brain by the application of the brandy? Is not the vascularity of the internal surface of the stomach somewhat increased, and the nervous structure excited? We do not say that the brandy produces these changes; but that the presence of the brandy causes the living structures to assume these changes themselves. This indeed appears to be the opinion of Dr. Chapman himself, for he says immediately afterwards—"the theory I shall propose of the operation of medicines alleges that they all act *by exciting a local impression*, which is extended through the medium of sympathy." Now we ask Dr. Chapman, what is this *impression*, if it is not some *change*, whether visible or invisible, of the parts so impressed or acted on? He says, in one page, that matters do *not* act on the healthy structure, and in the next page he says, they "excite a local impression." Surely this last expression conveys the idea of some agency in the material, and not a mere passiveness under the agency of the vital, structure. We defy the doctor, or any other man, to reconcile these two passages.

Our author denies that medicines enter the circulation and produce their effects in this way.

"All changes in the condition of the fluids are wrought by impressions made through the intervention of the solids. Not the slightest proof exists, so far as I know, of their undergoing any mutations, either by spontaneous action, or from the introduction of foreign matters, much less, that such is the cause of disease, or the mode in which remedies operate." 61.

This, we think, is carrying matters rather too far. We find actual changes in the blood and other fluids during life; and we really see no proof that they *always* take place through the agency of the solids. We do believe, however, that they generally take place through the said agency; but then we imagine that diseases may be the consequence of this change in the fluids, in whatever way the change is produced, whether by the living solids, or by spontaneous mutations in the fluids themselves. In this respect, therefore, we humbly beg leave to differ from Dr. Chapman.

Our author considers it quite incredible that any substance, after a subjection to the digestive and assimilative powers, should retain, in the slightest degree, its original properties. This, he thinks, is proved by the circumstance that the chyle has always an essential identity, however diversified may be the materials from which it is formed—that, in fact, it has probably all the properties of the blood, except its red colour. Three of the constituents of blood at least it contains.

"1. There is one portion of chyle, which preserves its fluidity during life, but coagulates after death, by exposure to the air, and is probably fibrine.

"2. There is a second portion, which resembles serum, in continuing fluid when exposed to the atmosphere, and in coagulating at the same degree of temperature, as serum.

"3. There is a third, consisting of globules, similar to those of blood, with this difference only, that they are much more minute." 62.

Even when substances are applied to the surface of the body and absorbed, the same mutation or assimilation, Dr. C. maintains, takes place as when they are introduced into the stomach and digested there. This capacity of the lymphatics, as well as of the lacteals, is a provision of nature to exclude noxious matters from the circulation. The obvious objection, that the properties of certain substances are displayed in the secretions, is thus attempted to be overcome.

"To me it is clear, that the process of assimilation, as performed either by the chylopoietic viscera, or by any part of the absorbent apparatus, completely decomposes all substances; and, however discrepant in their properties, reduces them to a homogenous fluid fitted for the purpose of nutrition. But, when thrown into the secretions or excretions, being removed beyond the control of the vital energies, chemical affinities are sometimes again brought into play, by which these substances are in part, or *wholly regenerated.*"

This work of *regeneration* appears to us a very lame petitio principii. Our author endeavours to strengthen it by pointing out to us that—

"Certain articles can only be detected in certain fluids, as the odour of garlic in milk, of asparagus in urine, of sulphur in the perspiration; and the colouring principle of madder is to be traced in no part of the solids, except the bones, and their immediate appendages, the cartilages. Did these articles pre-exist in the blood instead of being regenerated in some such manner as I have stated, ought they not to be thrown out indiscriminately by all the emunctories?"

To us the reason appears to be simply this—that some substances have a tendency to run to and affect one organ or emunctory, and others another. Our author objects to this explanation, because we cannot tell "why our remedies, after mixing with the blood, should be directed to one organ in preference to another." But the same objection surely lies against the doctrine of sympathy—why should a medicine *sympathetically* affect one organ in preference to another? we are not now arguing against the doctrine of sympathy, but shewing that its advocates have urged unreasonable objections against the humoralists. That medicines, in very many instances, act through the medium of the nerves rather than of the blood-vessels, we believe is now almost universally admitted. The action of wine, opium, digitalis, prussic acid, &c. offers ample illustration. But that some other substances enter the blood (modified no doubt) can hardly be denied after finding madder and mercury in the bones; and soda in excess in the blood of a person who had taken much of this medicine. Dr. Marcet has also shewn an immense quantity of iron in the bile of the knife-eater, as recorded in the Medico-Chirurgical Transactions.

Discarding the fluids then, in his enquiries relative to the operation of medicines, our author has recourse to that law of the animal economy termed sympathy, or consent of parts, for a solution of the problem.

"Conformably to the theory I have adopted, whenever a medicinal substance is applied to a susceptible portion of the body externally or internally, an action is excited, which is extended more or less, according to the diffusibility of the properties of the substance, or the degree of sympathetic connexion, which the part may maintain with the body generally. Thus a set of actions is raised, every one of which is precisely similar, provided they are confined to the same system, by which is to be understood parts of an identity of structure. If, however, the chain runs into other systems, it loses its homogeneous character, the actions being modified by the peculiar organization of the parts in which they may take place. These are principles of universal application. In every case, whether it respects the operation of remedies, or the production of disease, the spot primarily acted upon, is a point, from which is diffused the radiated impressions. This is a mode of action peculiar to living matter, and is remarkably distinguished from all other processes. An impression is made and extended without mixture or combination, or in any degree disturbing the order and constitution of the part in which it takes place. But in chemical operations, to which we must look for an explanation, in the event of rejecting the above rationale, decomposition inevitably occurs—and, as the result, new compounds must be formed from a union of the elements of the part, with the substance applied, wholly subversive of the existing structure. By a course of medicine, and, indeed, by every meal, our nature would become essentially changed. Exceedingly preposterous as it may seem, such is the direct and legitimate corollary, from any other than the sympathetic view of the modus operandi of agents on the animated frame." 71.

By this our author is not to be considered as denying, but as affirming the vitality of the blood. The animal machine he views as constituted of solids and fluids, the whole of the latter, whatever the different colours (not including the excretions) are considered as blood.

"It is a whole made up of parts, which, though somewhat dissimilar, and existing in different proportions, are no less endowed with the vital influence, and held together, and made to harmonize, by common susceptibilities and sympathies. Connected indissolubly by these ties, an impression made on any one portion of the system, whether solid or fluid, is equally felt by both, on account of this established consent of parts." 72.

Our author observes that there are not wanting men who affect scepticism as to the very existence of what is termed sympathy, because it cannot be demonstrated before their eyes. As well might they doubt the existence of sensibility or irritability, neither of which is capable of demonstration. Of this principle, like many other principles, we know nothing except from the experience of its effects—its precise nature being concealed—causa latet, vis est notissima. Dr. Chapman is of opinion that sympathy cannot be exclusively referred to the nerves, although in many instances it is clearly brought about through their agency.

"But there are many other sympathies, not less conspicuous, between parts, the nerves of which have not the slightest connexion. It appears that, either by the co-operation of different organs in the performance of a function, as in the complex apparatus subservient to respiration, or from similarity of structure, parts, though detached, being prone to be affected by the same cause, as the parotid gland and testes in the male, and the same gland with the mammæ in the female, the habit of acting in unison is acquired, and sometimes confirmed. This habit of concerted action is termed association, and has been adopted as a principle by Locke, by Hartley, and by Darwin, to account for the connexion in many of the motions of the body, as well as in the operations of the mind. Both the sound and morbid states of the system present numerous instances of these associated actions, some of which are constant and uniform, while others are occasional and anomalous, produced, as it were, accidentally." 75-76.

This principle, our author properly observes, pervades the whole body, every portion of it being susceptible of associative actions, by which means the several parts are linked together so as to constitute one whole. But though this general medium exists, for the reception and propagation of impressions, there are three surfaces on which remedies, and perhaps the causes of disease more particularly operate. These are, the almentary canal, the skin, the organ of smell. Although each of these parts possesses considerable susceptibility, and maintains extensive connexion with the general system, it is the stomach which evinces infinitely the quickest sensibility to action, and the most intimate and multiplied relations. Without a stomach no animal can exist, this viscus being the most indispensible of the vital organs, and incident to every variety and gradation of animal existence. This organ is probably the centre of associations, and the seat of the first link in the chain of most diseases—"and is always the chief medium of the operation of our remedies in the correction of morbid derangements." It is needless to say that the intestinal canal is a mere continuation of the stomach, thus giving a most extensive spread to the play of sympathies and associations.

In respect to the surface of the body, much discussion has taken place as to the capacity or incapacity for absorption. At one time it was supposed that the skin readily absorbed every thing applied to it; at another the power of absorption was denied almost in toto to this surface. Leguin in France, and Rousseau in America, were among the first and foremost asserters of the latter doctrine. It is now, however, admitted by the heads of the non-absorption party, that the skin is capable of taking up certain substances under certain circumstances. Dr. Massey, having clearly proved that if the body be immersed in a decoction of madder, the colouring matter of this substance will be taken in, and displayed in the urine, by using any one of the alkalies as a test, set Dr. Rosseau and his friends to work in order to prove or disprove this formidable fact. The result of their researches was:—

"1. That of all the substances employed, madder and rhubarb are those only which affect the urine—the latter of the two, the more readily enters the system. Neither of these articles can be traced in any other of the secretions or excretions, or in the serum of the blood.

"2. That the power of absorption is limited to a very small portion of the surface of the body. The only parts, indeed, which seem to possess it, are the spaces between the middle of the thigh and hip, and between the middle of the arm and shoulder.

"Topical bathing, with a decoction of rhubarb or madder, and poultices of these substances applied to the back, or abdomen, or sides, or shoulders, produced no change in the urine: Equally ineffectual was immersion of the feet and hands in a bath of the same materials, which, after being kept in it for several hours, not the slightest proof of absorption was afforded." 81.

Such being the statements of the non-absorbent party, it is evident that cutaneous absorption must be admitted in general terms; for it would be absurd to suppose (whatever experiments indicate to the contrary) that the power of absorption is limited to the skin "between the middle of the thigh and hip, and between the middle of the arm and shoulder." Neither can we doubt the fact of pulmonary absorption, notwithstanding the attempt of our ingenious author to account for the phenomenon by shewing "that, under such circumstances, it is the olfactory nerves which are the seat of the impression, and the medium through which these volatile matters produce their effects." The experiments to which the above passage has allusion are too numerous for us, at present, to notice, but, says our author—

"Collectively, they warrant the conclusion, that, by simply closing the nostrils, either by compression with the fingers, or by filling them up with putty, the fumes of ardent spirits, of a strong decoction of tobacco, or an infusion of opium, may be inhaled for one hour, without any unpleasant effects: whereas, if the precaution mentioned, be omitted, the consequences are proved to be most distressing.

"New as these results are, and inconsistent with our pre-existent notions as they may be, they are rendered highly probable, independently of the respectability of the source whence they proceed, by some facts of a very striking and indisputable nature. Every practitioner has witnessed, how powerfully all the volatile and odorous matters operate on the olfactory nerves in health and in sickness: and it is hardly less known, that when the sense of smell is impaired by a coryza, or entirely suspended by obstructing the nostrils, that the sensible qualities of most substances are so lost, that they cannot be accurately discriminated; and this extends, even to those articles of our food or drink, with which we are most familiar." 82-3.

The subject of the *modus operandi* of medicines will be again adverted to when particular medicines are under consideration. In respect to the classification of medicines, as of diseases, much difficulty has always been experienced; and all attempts have failed when the arrangement has been made according to the sensible qualities, chemical compositions, or botanical characters of medicinal substances. The most natural and useful arrangement is according to their effects on the living body—yet there are endless perplexities and contrarieties in consequence of the *diversified* powers of many medicines, and the varying susceptibility of the constitution in health and in disease. Our author then examines the materia medica under titles expressive of the actions or operations of medicines on the body, as emetics, cathartics, diaphoretics, &c.

1. *Emetics.* Dr. Chapman does not give a very decided opinion respecting the mooted physiology of the action of vomiting; but he seems to lean to the opinion of Haighton, in opposition to Majendie, that the stomach is not passive but active in this operation. It is remarkable, he observes, that whereas most other medicines lose their power by repetition, emetics increase the susceptibility of the stomach to their impression the oftener they are used, till at last the very sight of them will excite vomiting. Before entering on the direct application of this class of medicines to diseases, our author suggests the following general precepts to be attended to in their exhibition.

"1. When the vessels of the head are full, or there is much general plethora, the emetic should be preceded by the loss of blood. Two advantages result from this practice. It renders the vomiting safe, and more easy and effectual. By neglecting this admonition, many a life has been either endangered, or sacrificed, by apoplexy or hæmoptysis.

"2. When the necessity is urgent, and a certain and powerful operation is demanded, give a large dose, and of the most active species. But, in all ordinary cases, minister the medicine in divided quantities, so as to guard against too violent an effect.

"3. Where the object is to make a strong revolutionary impression on the system, little drink should be allowed. But if it be to evacuate the contents of the stomach, large draughts of tepid water, or some other light drink, as warm chamomile tea, will promote this end, and, at the same time, facilitate vomiting.

"5. As a general rule, emetics should be given on an empty stomach, and in the morning, acting then with greater certainty, and less distress. They will, however, answer very well in the evening, and, where an apprehension exists of the patient taking cold, ought to be exhibited to him in bed, at the hour of rest.

"6. To check inordinate vomiting, direct laudanum, combined with some cordial, apply fomentions to the pit of the stomach, and sinapisms to the extremities. Chicken water, copiously drank, is sometimes useful, by turning the action downwards. When these fail, anodyne injections may be resorted to, and a large blister should be put on, over the epigastric region." 102.

In this section, Dr. Chapman takes occasion to enunciate his creed respecting fever. The distinction between idiopathic and symptomatic fevers is considered absurd. All fevers, he asserts, are caused by either animal or marsh effluvia, or variations of temperature. We are rather surprized, that a man of Dr. Chapman's comprehensive views should limit his etiology of fever within such narrow bounds. There are more than a hundred different causes that may give rise to the phenomena of fever. We shall only allude to two or three in addition to our author's catalogue. Mental emotions, excessive exercise, excessive heat, (independent of variations of temperature) excessive eating or drinking, and numerous other agents and circumstances around us, are all capable, as Dr. Chapman himself afterwajds admits, of exciting fever.

Dr. C's reason for blotting out idiopathic fever from the nosological chart is, that the cause of the disease makes its impression on a single point or organ, and thence radiates to the whole system—in short, that the disease has "its origin in local irritation." The seat of this local irritation is, according to our author, the stomach, thus agreeing with Broussais, whose doctrines, Dr. Chapman affirms, he anticipated in his lectures. Now, we beg leave to differ from Dr. Chapman on this point. Of the mode in which marsh and animal poison gets into the system we know nothing, although

Dr. C. confidently asserts, that it *must be* by the saliva. It is needless to say, that he can offer us nothing in the shape of proof. On the other hand, there are many causes of fever which cannot enter by the mouth, as mental emotions, &c. and where we have more reason to *suppose* (for it can only be supposition) that the brain and nervous system receive the first impression. We agree with our author then, that the causes of fever make their first impressions locally, the locality being various, as the nervous system, stomach, skin, lungs, &c. but then, we maintain, that it is not *fever* till the other parts are drawn into disorder, which other parts often become more severely affected than the point or organ on which the original impression was made. What nonsense is it then, to call fever, when it is playing on all the organs and systems of the body, a local disease, merely because its cause was applied to a part of the body first! We might as well call variola a local disease because the virus was inserted in the arm.

Our author's theory (gastric) naturally leads him to speak of the utility of emetics in this class of diseases; and we join him in thinking we have too readily relinquished their employment in favour of purgatives. But, as he truly says, there is a fashion in medicine as in all other things, and this is one of those revolutions to which the practice of our art is exposed. The reputation of emetics is again reviving in America—especially in those sections where bilious fevers prevail.

"Exhibited early in such fevers, an emetic operating well will frequently check an attack, and, in the more advanced stages, judiciously repeated, we shall find by it the pulse reduced, pain in the head relieved, sickness of stomach appeased, temperature of the surface lowered, with diaphoresis, which restores quietness, and hastens a critical solution." 108.

Speaking of puerperal fever, and the clashing opinions which have been given of its nature, our author observes as follows:—

"My observations have taught me, that, in this species of puerperal fever, venesection is of indispensable utility, though only in the early stages of the attack, and that, on the whole, I have derived most advantage from it, aided by prompt and copious purging, the occasional use of an emetic, when clearly indicated; by fomentations, and emollient embrocations to the abdomen, and, in the more advanced period of the disease, from the regular exhibition of camphor alone, in large doses, or with opium and emetic tartar, and, finally, by the spirit of turpentine externally applied, and internally given in such quantities, as shall make a decided impression." 113.

Our author's general remarks on emetics, and also his remarks on the particular substances employed, are judicious, but not particularly novel to the profession.

2. *Cathartics.* Professor Chapman's observations under this head, are characterized by judgment, and indicate careful attention at the bed-side of sickness. We cannot advert but to a very few points of this section. Dr. C. is a strenuous advocate for the chylopoietic origin of gout, and for the practice of free purgation in that disease. He bewails the lethargy of the profession in respect to the treatment of gout, occasioned, he thinks, by the doctrines of Sydenham and Cullen.

"My impression," says he, "very concisely stated, is, that this disease, if not originating in, has a most intimate connection with, certain states of the alimentary canal. I am inclined to this view of the subject, among other reasons, from having so frequently observed gout to commence with those symptoms, which denote a depraved condition of the stomach and bowels. The principal indications of an approaching attack of the disease, are, almost invariably, flatulence, sour eructations, indigestion, depraved appetite, nausea, strong sensations of internal heat, and obstinate constipation, or laxity of the bowels."

"I have now, for many years, habitually employed purgatives in the paroxysms of gout, and with unequivocal advantage. Not content with simply opening the bowels, I completely evacuate, by active purging, the entire alimentary canal. This being accomplished, all the distressing sensations of the stomach which I have mentioned, are removed, the pain and inflammation of the limb gradually subside, and the paroxysm, thus broken, speedily passes away. To effect these purposes, however, it is often necessary to recur to the remedy repeatedly." 190.

In palsy, our author asserts, that he has derived far more success from free purging, than from the usual routine of bleeding, blistering, and stimulating embrocations, to which he formerly had recourse.

"Dissatisfied with this course, I have, for many years, abandoned it, and rely, now, almost exclusively, on evacuating the bowels, by the drastic purgatives. Of the propriety of the change, I can entertain no doubt, the success having exceeded my most sanguine expectations. To do justice to the practice, it should be steadily persisted in, and aided by such remedies, as the cases may, from time to time, demand. Of the auxiliary means to which I allude, there is none so important as a repetition of blisters, not to the affected limb, for this is comparatively useless, but to the back of the neck, or, what answers still better, caustic issues on the same part, or behind the ears, or on the top of the head. These drains must be kept freely discharging, by irritating dressings." 193.

In epilepsy, Dr. Chapman has had considerable success from purgatives, to which he was led by the total failure of the ordinary plans of treating the disease—his views coincide with those of Dr. Prichard, in placing the cause of epilepsy very frequently in the bowels. In respect to purgatives, however, "it will not do, (he says) merely to evacuate the bowels; cathartics must be repeated day after day, without interruption, unless absolutely forbid by circumstances." By continuing this course for several months successively, he has cured several cases of the disease completely, and afforded considerable relief in others. It is not to be understood that he confines his whole methodus medendi to purgation. Local and general bleeding, low living, and other auxiliaries are employed.

On the particular purgatives, our author is copious, and in general, correct in his observations, but we cannot follow him in this division of the subject. The following passage will give great offence to the anti-mercurial drivellers of this country, who seem to think, they are on the point of driving every preparation of mercury entirely out of the pale of practice.

> "Calomel, on every account, seems to be peculiarly adapted to the cases of children. Whether we wish to relieve actual disease, or merely to evacuate the contents of the bowels, it operates leniently and efficaciously. Yet, by many, it is supposed to be a very violent purgative, and, hence, there is a sort of popular prejudice against its use in the complaints of children, an error, which leads to so much mischief, that we ought to unite to remove it. From an extensive experience with the medicine, I am entirely convinced, that in those cases, its action is incomparably milder, than in more advanced life."
>
> "The dose of calomel for an adult, when taken alone, is from ten to twenty grains. We commit a mistake in giving too small a quantity of this medicine. Employed largely, its action is infinitely less irritating to the stomach and bowels—and is not so apt to be rejected by vomiting—its purgative operation being more prompt and complete. I have known a drachm to be taken at a time without inconvenience, and even without much increase of effect." 229.

We do not advocate the latter practice in this country, where cold and atmospheric vicissitudes are apt to interrupt the action of purgatives and render their effects more uncertain—consequently where the medicine would run sometimes to the mouth, when it did not pass freely through the bowels. Nevertheless we can vouch for the correctness of Dr. Chapman's statement, and for the propriety of his therapeutical observations, especially as they relate to the climate in which he lives, and other climates of a still hotter kind.

Speaking of Rhubarb, our author makes the following statement which, if confirmed by others, will prove a discovery of the greatest utility in the practice of medicine.

"It (rhubarb) has also this further peculiarity, that, however combined with opium, its purgative property is not at all restrained: and, hence, is invaluable in cases where a necessity for evacuating the bowels is connected with so much pain, as to demand the interposition of opiates. Of the accuracy of this observation, which, so far as I know, is original, I cannot permit myself to doubt, having confirmed it by ample experience." 231.

3. *Diuretics.* Our author's observations on this important class of medicinal agents are by no means uninteresting or uninstructive, especially to the junior classes of society. Dr. Chapman's views will not permit him to subscribe to the belief that articles of this class enter the circulation, with a retention of their powers, and act directly on the kidneys. We confess that the doctrine of sympathy has also its difficulties in explaining the modus operandi of diuretics. The discussion of the theoretical question does not, however, materially affect the practical application of the knowledge we possess on the subject. It is pretty generally agreed that an increase of the urinary secretion may take place either by stimulating the kidneys, or by invigorating the powers of absorption. It is also well known that the skin and urinary apparatus are vicarious in their action. On this account, in the administration of diuretics, we ought to avoid much external heat. The operation of this class of medicines is also well known to be greatly increased by plentiful dilution; and we believe all idea of danger from indulging in the latter, where dropsical effusion prevals, is now pretty nearly abandoned. The effect of diuretics is moreover promoted by the direct reduction of arterial action—especially by blood-letting, a fact that was well known for many years in this country and America, long before Majendie, by his late experiments, excited the attention of the profession to this point.*

We agree with Dr. Chapman, and with many of the ancient writers, that the urinary secretion is a more important agent both in health and in disease than modern physicians seem to allow.

---

* Our tropical writers have long ago shewn that, in the grave fevers of those climates, mercury would not be absorbed, nor purgatives act till copious depletion by the lancet had taken place. Yet Majendie is lauded to the skies for *discovering* in dogs what had long been discovered in man!

"The kidneys are one of the emunctories, through which nature, when oppressed by disordered action, endeavours to relieve herself, and this she does chiefly, by throwing off the more watery parts of the blood, which, in some instances, amount to a very large quantity. Evacuations of this kind, by emptying the blood vessels, though, perhaps, not so effectual, have, unquestionably, a tendency to reduce morbid excitement, and, therefore, are entitled to be classed with the other depleting remedies, as venesection, sweating, purging, &c." 266.

Of the particular diuretics it will not be in our power to take any material notice. It appears that Dr. Chapman has been in the habit of administering the bals. copaibæ in the first stages of gonorrhœa, for twenty years past, and experience, he says, has increased his confidence in the remedy. The following is one of the forms which he has found to agree best with the stomach.

℞. Bals. Copaib.

Spir. æth. nit. āā ℥ss

Albumen. ovi—

Sacchari alb. āā ʒi

Aquæ fontanæ—℥iij. ℳ ft. mistura, cujus capiat cochleare unum mag. ter die.

In the treatment of gonorrhœa he strongly insists on one particular caution—rigid abstinenceand perfect repose. To our knowledge these *alone* will cure nine cases out of ten of gonorrhœa in a shorter space of time than any other remedy in the materia medica.

The squill is a favourite medicine with Dr. Chapman in dropsical effusions of the chest—a disease very common in America. He exhibits one grain of squill and three of calomel, night and morning.

"When calomel purges unduly, which it will sometimes do, we may restrain it, by adding to the preparation a little opium. In the use of mercury, the mouth becoming affected, is always an auspicious circumstance. I have observed, especially in hydrothorax, that the distressing symptoms commonly subside on the appearance of ptyalism, which is not altogether owing to the mercurial action, since mercury alone will not so often produce the same effect," 300.

IV. In the section on lithontriptics we do not see any thing that need detain us, unless it be the following passage.

"The relation which the terebinthinates bear to the urinary organs, is well established. But in no case do they display such valuable powers, as in the connection of the lithic diathesis and in the alleviation of the pain of the nephritic paroxysm. Of the different preparations of turpentine, the oil is to be preferred for these purposes, and its effects are often very extraordinary. I have known a single dose to put an end to the deposition of red sand, and, at the same time, relieve the most acute suffering, with the promptness of an opiate. On what principle it operates is not very intelligible, though it would seem that its appearance in the bladder is necessary to its success. I am told by Dr. Physick, to whom I owe much of the information which I possess on the subject of this article, that whenever it has failed with him, the *violet odour* was wanting in the urine.

"The oil of turpentine may be directed in these cases, in the dose of from ten to twenty drops, several times a day, and the best mode of exhibition, is on a tea spoonful of powdered sugar, to be washed down with a little water." 343.

V. *Diaphoretics.* This section contains much judicious observation and sound reasoning. Speaking of the good effects of diaphoretics in bowel complaints, our author makes the following remark—a remark which our readers know we have often made in this journal as well as elsewhere.

"It has long been my conviction, that, in the bowel affections, we have, as a general rule, *purged infinitely too much.* Cherishing, still, the antiquated notion of morbid humours, it is usual, in these complaints, with some practitioners, to evacuate the intestines, as long almost as any discharge can be procured, under the impression, that the matter retained is irritating and offensive, and, therefore, the immediate source of all the mischief. *The very reverse of this, I hold to be true.* It appears to me, that the accumulation of acrid matter, is the *effect of previous irritation* in the stomach and bowels, which causes an increased effusion from the mucous follicles, or the exhalent vessels, and, sometimes, a very vitiated secretion of bile. Deducing my practice from this view of the nature of the disease, I have been accustomed, after comparatively moderate evacuations, to exhibit medicines so compounded, as to meet the double indication of allaying intestinal irritation, and, more remotely, of relaxing the surface. Combinations of opium and ipecacuanha, to which calomel may sometimes be added, are an invaluable preparation for this purpose. But the irritation being excessive, and, as usual, productive of frequent and painful discharges, I either augment the quantity of opium, or, what is more effectual, administer anodyne injections, three or four times in the course of twenty-four hours. These remedies will, in most cases, calm the irritation of the bowels, and, as soon as this happens, the acrid discharges, together with the other symptoms, cease to be troublesome." 365.

Speaking of the particular diaphoretics, our author has occasion to mention sulphur, both as an internal and external application, and states the following fact.

"On this subject, I shall further observe, that I have found nothing so speedily to cure tinea capitis, as an ointment made of an ounce of sulphur and lard each, with an addition of two drachms of sal. ammoniac. No cutaneous disease is more difficult to manage, than this species of eruption is sometimes. I have known cases of it to baffle the united skill of some of the ablest practitioners. But, since using the above ointment, I have been uniformly successful." 392.

We have now closed the first volume of the work before us, and as it would extend this article too far to notice the second volume, we shall defer the analysis of it till our next number. Our opinion of Dr. Chapman's publication is decidedly favourable, as may easily be gathered from the general tenor of this article.

